# Information Wanted—Reports of Typhoid-Vaccine Cases

**Published:** 1912-10

**Authors:** 


### Information Wanted—Reports of Typhoid-Vaccine Cases.

*To the Readers of the Texas Medical Journal:*

About six years ago the writer began to use vaccines in the treatment of typhoid fever. Since that time he has thus treated more than one hundred cases and has obtained numerous articles upon the same subject written by physicians in various parts of the world. It seems possible, however, that some may have escaped notice. He also realizes that many of the profession may have treated some cases without reporting them. A paper upon the subject is now in the course of preparation. In this it is earnestly desired to incorporate reports from a large number of cases—good, bad, and otherwise. He accordingly makes the following request to the readers of this JOURNAL:

Will anyone who has used vaccines in the treatment of typhoid fever, whether but one case or more, kindly communicate to him that fact, accompanied by name and address of the reporter? If the results have already been reported, a note of the journal in which they appeared will be sufficient. If they have not been reported, a short blank form will be sent to the physician to be filled out. Due credit will be given in the article to each person making a report. If any physician happens to know of other confreres who have any such cases, it will be appreciated if he sends their names, as they may not happen to read this note. It is hoped that by this means a sufficient number of cases may be collected to somewhat definitely settle the now mooted question whether vaccines are or are not of benefit in typhoid therapy.

Reports of cases will be accepted at any time in the future but preferably by November or December of the present year.

Kindly communicate with Dr. W. H. Watters, Director of the Department of Pathology and Bacteriology, Evans Institute for Clinical Research, Boston, Mass.

